# Supplementary material for: Pyruvate Dehydrogenase Kinase 1 inhibition mediated oxidative phosphorylation enhancement in cartilage promotes osteoarthritis progression
Source: BMC Musculoskelet Disord. 2023 Jul 20;24:597. doi: 10.1186/s12891-023-06585-6 (PMC10357736; doi:10.1186/s12891-023-06585-6)
Supplement: Supplementary file 1 — Additional file 1. [file 12891_2023_6585_MOESM1_ESM.zip › BP.Gsea.1653623667859/GOBP_POSITIVE_REGULATION_OF_PROTEIN_DEPOLYMERIZATION.html]

Details for gene set GOBP\_POSITIVE\_REGULATION\_OF\_PROTEIN\_DEPOLYMERIZATION[GSEA]

|  || Dataset | 55457\_collapsed\_to\_symbols.cls.cls #OA\_versus\_normal.cls.cls #OA\_versus\_normal\_repos |
| Phenotype | cls.cls#OA\_versus\_normal\_repos |
| Upregulated in class | OA |
| GeneSet | GOBP\_POSITIVE\_REGULATION\_OF\_PROTEIN\_DEPOLYMERIZATION |
| Enrichment Score (ES) | 0.47655192 |
| Normalized Enrichment Score (NES) | 1.4304818 |
| Nominal p-value | 0.08609272 |
| FDR q-value | 0.45105213 |
| FWER p-Value | 1.0 |
Table: GSEA Results Summary

  

Fig 1: Enrichment plot: GOBP\_POSITIVE\_REGULATION\_OF\_PROTEIN\_DEPOLYMERIZATION      
 Profile of the Running ES Score & Positions of GeneSet Members on the Rank Ordered List

  

| SYMBOL | TITLE | RANK IN GENE LIST | RANK METRIC SCORE | RUNNING ES | CORE ENRICHMENT || 1 | KATNB1 | katanin regulatory subunit B1 [Source:HGNC Symbol;Acc:HGNC:6217] | 158 | 0.852 | 0.1798 | Yes |
| 2 | STMN2 | stathmin 2 [Source:HGNC Symbol;Acc:HGNC:10577] | 353 | 0.723 | 0.3277 | Yes |
| 3 | SPAST | spastin [Source:HGNC Symbol;Acc:HGNC:11233] | 1502 | 0.429 | 0.3352 | Yes |
| 4 | WDR1 | WD repeat domain 1 [Source:HGNC Symbol;Acc:HGNC:12754] | 1851 | 0.379 | 0.3937 | Yes |
| 5 | SLN | sarcolipin [Source:HGNC Symbol;Acc:HGNC:11089] | 2510 | 0.302 | 0.4107 | Yes |
| 6 | ACTN2 | actinin alpha 2 [Source:HGNC Symbol;Acc:HGNC:164] | 2532 | 0.299 | 0.4766 | Yes |
| 7 | SEMA5A | semaphorin 5A [Source:HGNC Symbol;Acc:HGNC:10736] | 3308 | 0.220 | 0.4661 | No |
| 8 | PLEK | pleckstrin [Source:HGNC Symbol;Acc:HGNC:9070] | 4382 | 0.122 | 0.4104 | No |
| 9 | VIL1 | villin 1 [Source:HGNC Symbol;Acc:HGNC:12690] | 4948 | 0.076 | 0.3837 | No |
| 10 | WASHC2C | WASH complex subunit 2C [Source:HGNC Symbol;Acc:HGNC:23414] | 7471 | -0.116 | 0.2141 | No |
| 11 | NES | nestin [Source:HGNC Symbol;Acc:HGNC:7756] | 7822 | -0.143 | 0.2191 | No |
| 12 | DSTN | "destrin, actin depolymerizing factor [Source:HGNC Symbol;Acc:HGNC:15750]" | 7878 | -0.146 | 0.2479 | No |
| 13 | TRPV4 | transient receptor potential cation channel subfamily V member 4 [Source:HGNC Symbol;Acc:HGNC:18083] | 7925 | -0.149 | 0.2779 | No |
| 14 | F2RL1 | F2R like trypsin receptor 1 [Source:HGNC Symbol;Acc:HGNC:3538] | 8631 | -0.203 | 0.2689 | No |
| 15 | CARMIL1 | capping protein regulator and myosin 1 linker 1 [Source:HGNC Symbol;Acc:HGNC:21581] | 9529 | -0.276 | 0.2616 | No |
Table: GSEA details [plain text format]

  

Fig 2: GOBP\_POSITIVE\_REGULATION\_OF\_PROTEIN\_DEPOLYMERIZATION      
 Blue-Pink O' Gram in the Space of the Analyzed GeneSet

  

Fig 3: GOBP\_POSITIVE\_REGULATION\_OF\_PROTEIN\_DEPOLYMERIZATION: Random ES distribution      
 Gene set null distribution of ES for **GOBP\_POSITIVE\_REGULATION\_OF\_PROTEIN\_DEPOLYMERIZATION**
